# Rural-urban Disparities in the Prevalence of Mild Cognitive Impairment and Dementia in Taiwan: A Door-to-door Nationwide Study

**DOI:** 10.2188/jea.JE20200602

**Published:** 2022-11-05

**Authors:** Chih-Ching Liu, Chien-Hui Liu, Yu Sun, Huey-Jane Lee, Li-Yu Tang, Ming-Jang Chiu

**Affiliations:** 1 Department of Healthcare Administration, College of Medical and Health Science, Asia University, Taichung, Taiwan; 2 School of Nursing, National Yang Ming Chiao Tung University, Hsinchu, Taiwan; 3 Department of Neurology, En Chu Kong Hospital, New Taipei City, Taiwan; 4 Taiwan Alzheimer’s Disease Association, Taipei, Taiwan; 5 Department of Neurology, National Taiwan University Hospital, College of Medicine, National Taiwan University, Taipei, Taiwan; 6 Graduate Institute of Brain and Mind Sciences, College of Medicine, National Taiwan University, Taipei, Taiwan; 7 Graduate Institute of Psychology, College of Science, National Taiwan University, Taipei, Taiwan; 8 Graduate Institute of Biomedical Electronics and Bioinformatics, National Taiwan University, Taipei, Taiwan

**Keywords:** dementia, mild cognitive impairment, prevalence, risk factors, urbanization

## Abstract

**Background:**

Screening or diagnosis for the elderly with dementia in rural regions might be delayed and underestimated due to limited utilization of healthcare resources. This study aimed to evaluate the disparities of prevalence and risk factors of mild cognitive impairment (MCI) and dementia between urban and rural residence.

**Methods:**

In this nationwide door-to-door survey, 10,432 participants aged 65 years and more were selected through computerized random sampling from all administrative districts in Taiwan and were assessed using an in-person interview. We calculated the prevalence of MCI and dementia, with their risk factors examined using multivariable logistic regression.

**Results:**

The prevalence of dementia in rural, suburban, and urban areas among the elderly was 8.69% (95% CI, 8.68–8.69), 6.63% (95% CI, 6.62–6.63), and 4.46% (95% CI, 4.46–4.47), respectively. A similar rural-suburban-urban gradient relationship on the dementia prevalence was observed in any age and sex group. The rural:urban ratio was higher in women than in men for both MCI and dementia. Urbanization remained to be an independent factor for both MCI and dementia after adjustment for age, gender, education, lifestyle, and health status. The beneficial effects of exercise on dementia were more evident in rural areas than in urban ones.

**Conclusion:**

Significantly higher prevalence of MCI and dementia were found in rural areas than in urban ones, especially for women. The odds of risk factors for MCI and dementia varied by urbanization status. Focus on the rural-urban inequality and the modification of associated factors specifically for different urbanization levels are needed.

## INTRODUCTION

Dementia has become a global public health priority, as it is closely associated with considerable consequences, such as disability, mortality, and socioeconomic burden.^1^^,^^2^ People with mild cognitive impairment (MCI) are recognized as a high-risk group for developing dementia.^3^ As global aging continues, coupled with rising urbanization, understanding of the association between urbanization and the prevalence of MCI and dementia among older adults is essential for adequate planning of public health interventions and rational allocation of health resources. Many previous studies have explored the role of urbanization in older adults’ risk of MCI^4^^–^^7^ and dementia.^7^^–^^16^ However, these results were inconclusive. Some studies showed higher prevalence rates of MCI and dementia in rural areas than in urban ones,^7^^–^^9^^,^^11^^,^^14^^,^^16^ while other studies reported opposite findings.^4^^–^^6^^,^^10^^,^^12^^,^^13^^,^^15^ Difference in lifestyle (eg, social participation,^17^ physical activity^18^), sociodemographic variables (eg, education level^11^^,^^16^^,^^19^), comorbidities (eg, cardiovascular disease^20^^,^^21^), accessibility to health care,^13^ and environmental factors (eg, pollutants/oxidative stress,^22^^,^^23^ pesticides^24^) may be responsible for these diversities. Besides, data and methodological challenges remain in the research on urbanization with MCI and dementia. Most previous studies on this issue used regional data without national representativeness,^5^^,^^6^^,^^8^^,^^10^^,^^12^^,^^13^^,^^15^^,^^16^ and were conducted in Western countries.^4^^,^^6^^,^^9^^,^^10^^,^^12^^–^^14^^,^^16^ Asian studies were still limited.^5^^,^^7^^,^^8^^,^^11^^,^^15^

In the current study, we explored the association between the degree of urbanization and the prevalence of dementia and MCI using a nationwide survey in Taiwan. Additionally, given rural-urban differences in many aspects, such as lifestyle, education, and comorbidities, we also examined whether MCI and dementia’s risk factors varied between urban and rural populations.

## METHODS

### Study design and sampling

This nationwide, population-based, cross-sectional survey in 19 counties or cities across the country was conducted from December 2011 through March 2013. The details of the survey sampling design, attrition, response rates, and data quality were described in a previous report.^25^ In brief, using a computerized multistage sampling design, we recruited nationally representative samples aged 65 years and older in urban and rural areas. With the assistance of the Ministry of Health and Warfare of Taiwan and local city governments, participants’ residential address was obtained to conduct a door-to-door survey. After participants provided written informed consent, an in-person interview was then performed to take a brief history related to cognitive and functional status, followed by a structured questionnaire with mental tests and demographic information, including sociodemographic data, lifestyle habits, medical comorbidities, and mental tests. All interviewers were well-trained in basic knowledge of dementia, diagnostic criteria, cognitive function measurements, and interviewing skills. The interview process was conducted based on an operational manual that defines all variables examined in this questionnaire. The lifestyle habits should be developed before the onset of dementia. Information about the duration (years) of the habits was also recorded. The detailed definitions of the lifestyle habits, including smoking, drinking, exercise, and social activity, were described in a published report.^26^ In brief, exercise was defined as physical activities persisting for at least 20 minutes to intensity capable of making one sweat. “Regular exercise” indicated the frequency was at least once per week. “Active and regular social activity” occurred at least once per week, including attending clubs or social groups, engagement in religious activities, meeting friends and family or others. Smoking and drinking were considered as habits if they occurred three or more times per week. Comorbid diseases of our participants were also evaluated and reported in another article.^27^ Most common and dementia-related comorbidities among the elderly, including hypertension, diabetes mellitus, cerebrovascular disease, cancer, and head injury, were analyzed in the current study. We performed logic checks for inconsistency and auditing to ensure the entered data’s reliability and quality.^25^ The Ethics Committee of the National Taiwan University Hospital approved the study protocol (DOH100-TD-M-113-100001).

### Case ascertainment

Participants with all-cause dementia or MCI or normal cognition were identified. Cognitive status was determined from an in-person evaluation. The diagnosis for all-cause dementia and MCI was based on the core clinical criteria recommended by the National Institute on Aging Alzheimer’s Association.^28^^,^^29^ A brief medical history was taken from the participant and a knowledgeable informant. Objective mental assessments included the Clinical Dementia Rating Scale, which determined the severity of dementia, and the Taiwanese Mental State Examination, which was taken to assess memory, language function, executive function, and visual-spatial ability. The inter-rater reliability of Clinical Dementia Rating Scale was substantial, as demonstrated by a kappa value of 0.67.^25^ Normal results from Taiwanese Mental State Examination were determined as a score >24 for literate older people and >13 for illiterate older people.^30^^,^^31^ Functional status was measured using activities of daily living and instrumental activities of daily living scale. The details of diagnostic criteria and the process of case ascertainment of this program were described in our previously published reports.^25^^–^^27^

### Levels of urbanization

We classified each subject’s living area at the interview time into three degrees of urbanization (urban, suburban, and rural). The urbanization classification was proposed by Huang et al.^18^ They categorized all 358 cities and townships of Taiwan into five ordered levels of urbanization based on different indicators, including the number of residents, population density, and the percentage of people working in secondary and tertiary industries.^18^^,^^32^^,^^33^

### Statistical analysis

The differences in the levels of urbanization in study participants were examined using a chi-squared test. Estimates of MCI and dementia prevalence in urban, suburban, and rural areas were analyzed for overall, age-stratified, and sex-stratified populations separately. The crude prevalence rates were calculated by dividing the number of dementia or MCI by the total number of people in this survey. We further calculated age- and sex-standardized prevalence rates of dementia or MCI using the World Health Organization 2000 standard population.^34^ Rural:urban prevalence ratios with 95% confidence intervals (CI) were computed to compare the prevalence in rural areas with that in urban areas. To evaluate the independent effects of urbanization on MCI or dementia prevalence, we conducted multivariate logistic regression analysis with adjustment for age, sex, education, lifestyle habits, and comorbidities.

Additionally, multivariate logistic regression was also used to examine the odds ratio of these variables on the prevalence of MCI or dementia between urban and rural populations. Given that social activity may correlate with other lifestyle factors in the cultural context, we assessed the interaction terms of social activity with smoking, alcohol intake, and exercise in the multivariable model. We also compared the odds ratios of these variables among the urbanization levels using a test for heterogeneity, which was quantified using the Cochran Q statistic and I-squared test. A *P* value <0.10 and I-squared >50% were considered to be significantly heterogeneous. The statistical analysis was performed using SAS version 9.4 (SAS Institute, Cary, NC, USA). A *P* value <0.05 was considered statistically significant.

## RESULTS

### Characteristics of the sampled populations

Of the 28,600 randomly sampled, elderly subjects, 10,432 (52% female) have completed the survey, of which 2,624 resided in urban areas, 3,693 in suburban areas, and 4,115 in rural areas. The mean age was highest in urban areas with 0.4 years older than those in rural areas. More people have a high educational level, regular exercise habit, and social activity in the urban areas. The percentages of hypertension, stroke, head injury, and cancer were also higher in urban areas than suburban and rural areas (Table 1).

**Table 1.** Characteristics by levels of urbanization (*N* = 10,432)

Variables	Urbanization level							

	Urban (*n* = 2,624)		Suburban (*n* = 3,693)		Rural (*n* = 4,115)		χ^2^	*P* value
		
	*n*	%	*n*	%	*n*	%		
Sex								
Men	1,244	47.4	1,755	47.5	1,975	48.0	0.28	0.870
Women	1,380	52.6	1,938	52.5	2,140	52.0		
Age, years								
65–69	504	19.2	649	17.6	700	17.0	71.17	<0.001
70–74	712	27.1	1,086	29.4	1,146	27.9		
75–79	561	21.4	863	23.4	1,050	25.5		
80–84	428	16.3	625	16.9	796	19.3		
≥85	419	16.0	470	12.7	423	10.3		
Mean (SD)	76.5 (7.3)		76.2 (6.8)		76.1 (6.3)			
Education, years								
0	470	17.9	1,130	30.6	1,752	42.6	968.37	<0.001
≤6	1,132	43.1	1,620	43.9	1,967	47.8		
7–12	680	25.9	662	17.9	319	7.8		
>12	342	13.1	281	7.6	77	1.9		
Lifestyle habits								
Smoking	524	19.9	734	19.9	746	18.1	5.13	0.077
Drinking	334	12.7	484	13.1	443	10.8	11.39	0.003
Regular exercise	1,258	47.9	1,517	41.1	1,345	32.7	162.08	<0.001
Social activity	1,136	43.3	1,118	30.3	1,142	27.8	189.78	<0.001
Comorbidities								
Hypertension	1,409	53.7	1,908	51.7	2,021	49.1	14.04	<0.001
Diabetes mellitus	580	22.1	804	21.8	864	21.0	1.33	0.515
Stroke	208	7.9	227	6.2	259	6.3	9.23	<0.001
Head injury	171	6.5	136	3.7	100	2.4	72.10	<0.001
Cancer	152	5.8	176	4.8	157	3.8	14.31	<0.001

SD, standard deviation.

### Prevalence of MCI and dementia

**Figure 1.**
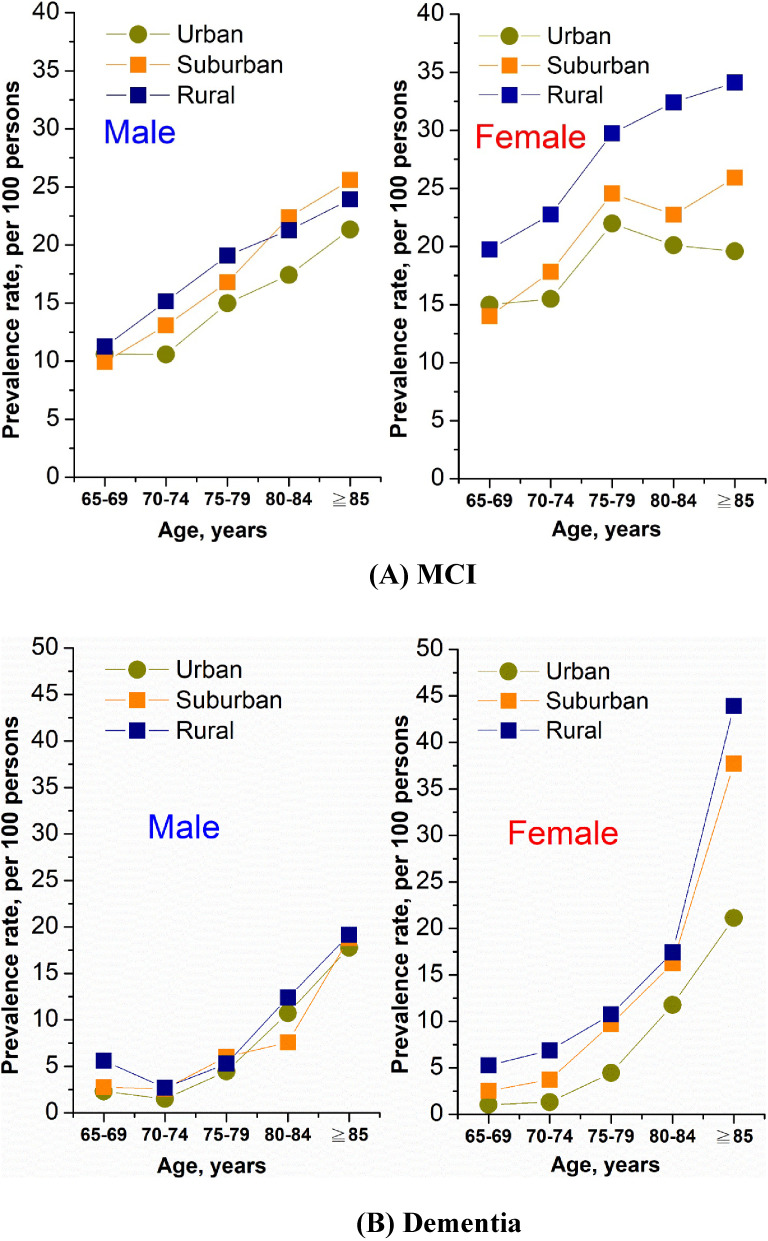
Age- and sex-specific prevalence rates of (A) mild cognitive impairment and (B) dementia by levels of urbanization (*N* = 10,432)

The standardized prevalence of MCI in rural, suburban and urban areas among the elderly over 65 years old was 20.29% (95% CI, 20.28–20.29%), 16.67% (95% CI, 16.66–16.67%), and 15.11% (95% CI, 15.11–15.12%), respectively, with a rural:urban ratio of 1.34 (95% CI, 1.27–1.41). As for dementia, the age- and sex-adjusted prevalence was also highest in rural (8.69%; 95% CI, 8.68–8.69%) and lowest in urban areas (4.46%; 95% CI, 4.46–4.47%) with a rural:urban ratio of 1.95 (95% CI, 1.78–2.13) (Table 2). A similar rural-suburban-urban gradient relationship was observed in both men and women, and in most of the age groups in terms of the prevalence of MCI and dementia. The differences in the prevalence of MCI between rural, suburban and urban areas were more evident in women than in men across all age groups, with the rural:urban ratio ranging from 1.32 at age 65–69 to 1.74 at age over 85 in women, while the ratios in the corresponding age groups were 1.06 and 1.12 in men (eTable 1). Regarding the prevalence of dementia, the effect of urbanization with higher rural:urban ratio in women than in men was even more significant (Figure 1 and eTable 1). For the elderly aged 65–69 and 70–74, the rural:urban ratios were up to 5.04 and 6.59 in women, while the corresponding ratios were 2.44 and 1.78 in men.

**Table 2.** Overall, sex-specific, and age-specific prevalence of mild cognitive impairment and dementia by levels of urbanization, Taiwan, 2011–2013

Variables	Crude rates, % (95% CI)			Rural/Urban ratio	Standardized rates,^a^ % (95% CI)			Rural/Urban ratio
	
	Urban	Suburban	Rural		Urban	Suburban	Rural	
Mild cognitive impairment								
Overall	16.46 (16.44–16.49)	18.74 (18.72–18.76)	22.48 (22.46–22.50)	1.37 (1.22–1.53)	15.11 (15.11–15.12)	16.67 (16.66–16.67)	20.29 (20.28–20.29)	1.34 (1.27–1.41)
Sex								
Men	14.63 (14.57–14.69)	16.87 (16.82–16.91)	17.72 (17.68–17.76)	1.21 (1.01–1.45)	12.93 (12.92–12.93)	14.55 (14.54–14.55)	15.75 (15.74–15.76)	1.22 (1.12–1.32)
Women	18.12 (18.06–18.17)	20.43 (20.39–20.47)	26.87 (26.83–26.91)	1.48 (1.28–1.72)	17.29 (17.29–17.30)	18.79 (18.78–18.80)	24.82 (24.81–24.83)	1.44 (1.30–1.58)
Age, years								
65–69	13.10 (12.96–13.23)	12.17 (12.07–12.27)	15.86 (15.75–15.96)	1.21 (0.89–1.64)	12.79 (12.78–12.80)	11.97 (11.96–11.98)	15.49 (15.48–15.51)	1.21 (1.10–1.33)
70–74	13.20 (13.11–13.30)	15.65 (15.59–15.72)	19.28 (19.22–19.35)	1.46 (1.15–1.86)	13.03 (13.01–13.04)	15.45 (15.44–15.47)	18.95 (18.93–18.96)	1.45 (1.31–1.62)
75–79	18.89 (18.76–19.03)	20.97 (20.88–21.07)	24.38 (24.30–24.46)	1.29 (1.03–1.62)	18.48 (18.45–18.50)	20.68 (20.65–20.71)	24.42 (24.39–24.45)	1.32 (1.18–1.48)
80–84	18.69 (18.51–18.87)	22.56 (22.43–22.69)	26.88 (26.78–26.99)	1.44 (1.11–1.86)	18.75 (18.71–18.80)	22.56 (22.52–22.61)	26.84 (26.79–26.89)	1.43 (1.25–1.64)
≥85	20.53 (20.34–20.71)	27.66 (27.47–27.85)	29.08 (28.87–29.29)	1.42 (1.08–1.87)	20.46 (20.39–20.53)	28.26 (28.19–28.34)	29.02 (28.94–29.09)	1.42 (1.20–1.67)

Dementia								
Overall	6.55 (6.54–6.57)	8.80 (8.79–8.82)	10.50 (10.48–10.51)	1.60 (1.34–1.91)	4.46 (4.46–4.47)	6.63 (6.62–6.63)	8.69 (8.68–8.69)	1.95 (1.78–2.13)
Sex								
Men	6.83 (6.79–6.87)	6.61 (6.58–6.64)	7.54 (7.52–7.57)	1.10 (0.85–1.44)	4.54 (4.53–4.54)	5.01 (5.01–5.02)	6.51 (6.50–6.51)	1.43 (1.26–1.64)
Women	6.30 (6.27–6.34)	10.78 (10.75–10.82)	13.22 (13.19–13.26)	2.10 (1.65–2.67)	4.39 (4.38–4.39)	8.24 (8.23–8.25)	10.87 (10.86–10.88)	2.48 (2.12–2.89)
Age, years								
65–69	1.59 (1.54–1.64)	2.62 (2.57–2.57)	5.43 (5.37–5.49)	3.42 (1.60–7.33)	1.67 (1.67–1.68)	2.63 (2.63–2.64)	5.44 (5.44–5.45)	3.25 (2.60–4.07)
70–74	1.40 (1.37–1.44)	3.22 (3.19–3.25)	4.97 (4.94–5.01)	3.54 (1.81–6.93)	1.41 (1.40–1.41)	3.18 (3.17–3.18)	4.79 (4.78–4.80)	3.39 (2.56–4.50)
75–79	4.46 (4.38–4.53)	8.00 (7.93–8.06)	8.00 (7.95–8.05)	1.80 (1.31–2.47)	4.46 (4.44–4.47)	7.86 (7.84–7.87)	8.02 (8.00–8.04)	1.80 (1.46–2.22)
80–84	11.21 (11.07–11.36)	11.84 (11.74–1.94)	14.95 (14.86–15.04)	1.33 (0.95–1.86)	11.24 (11.21–11.27)	11.90 (11.87–11.94)	14.93 (14.89–14.97)	1.33 (1.11–1.59)
≥85	19.33 (19.15–19.52)	27.66 (27.47–27.85)	31.68 (31.46–31.89)	1.64 (1.24–2.16)	19.46 (19.39–19.52)	28.26 (28.19–28.34)	31.53 (31.46–31.61)	1.62 (1.38–1.91)

CI, confidence interval.

^a^Standardized prevalence rates were calculated per 100 persons. The overall standardized rates were adjusted for age and sex, and standardized rates of men and women were adjusted for age, while the rates of each age group were adjusted for sex.

### Logistic analysis

Because the lower prevalence of MCI and dementia was noted as the urbanization increased, we calculated the odds ratio (OR) of urbanization status after adjustment for confounding factors, including age, sex, education, lifestyle habits, and comorbidities. Compared to residents in urban areas, people living in the suburban and rural areas were at significantly increased risk of dementia with an adjusted OR of 1.43 (95% CI, 1.13–1.81) and 1.73 (95% CI, 1.37–2.19), respectively (Table 3). For other risk factors, we found social activity has interactive effects with regular exercise in terms of the link with MCI, and both exercise and social activity were significantly associated with reduced prevalence for MCI and dementia. Female, old age, low education levels, and comorbidities, including diabetes, stroke, and head injury, were all associated with increased prevalence of both MCI and dementia (Table 3).

**Table 3.** Logistic regression model assessing the independent effects of urbanization, sociodemographic characteristics, lifestyle habits, and comorbidities on mild cognitive impairment and dementia

Variables	Mild Cognitive Impairment				Dementia			
	
	Crude OR	95% CI	Adjusted OR^a^	95% CI	Crude OR	95% CI	Adjusted OR^a^	95% CI
Levels of Urbanization								
Urban areas	1.00		1.00		1.00		1.00	
Suburban areas	1.21^b^	1.06–1.38	1.07	0.92–1.23	1.42^b^	1.17–1.73	1.43^b^	1.13–1.81
Rural areas	1.55^b^	1.36–1.76	1.17^b^	1.01–1.35	1.82^b^	1.51–2.19	1.73^b^	1.37–2.19
Sex								
Men	1.00		1.00		1.00		1.00	
Women	1.54^b^	1.39–1.70	1.25^b^	1.10–1.43	1.73^b^	1.50–1.99	1.50^b^	1.22–1.83
Age, years								
65–69	1.00		1.00		1.00		1.00	
70–74	1.24^b^	1.05–1.46	1.08	0.91–1.28	1.06	0.77–1.46	0.95	0.68–1.34
75–79	1.90^b^	1.61–2.24	1.53^b^	1.29–1.82	2.53^b^	1.88–3.40	2.19^b^	1.60–3.00
80–84	2.36^b^	1.98–2.81	2.07^b^	1.73–2.48	5.31^b^	3.98–7.09	4.96^b^	3.63–6.77
≥85	3.76^b^	3.11–4.56	3.42^b^	2.79–4.19	15.98^b^	12.00–21.28	16.56^b^	12.06–22.74
Education, years								
0	4.46^b^	3.46–5.76	3.49^b^	2.64–4.61	4.44^b^	3.08–6.40	2.32^b^	1.48–3.65
≤6	1.70^b^	1.32–2.20	1.59^b^	1.21–2.08	1.76^b^	1.22–2.54	1.53	0.99–2.38
7–12	1.05	0.79–1.40	1.08	0.81–1.46	1.14	0.76–1.72	1.12	0.69–1.82
>12	1.00		1.00		1.00		1.00	
Lifestyle habits								
Smoking (Ref. = No)	0.85^b^	0.75–0.97	1.10	0.94–1.30	0.63^b^	0.51–0.76	0.87	0.66–1.03
Drinking (Ref. = No)	0.78^b^	0.67–0.92	0.99	0.82–1.19	0.56^b^	0.42–0.70	0.80	0.58–1.10
Regular exercise (Ref. = No)	0.51^b^	0.46–0.57	0.64^b^	0.58–0.72	0.14^b^	0.11–0.17	0.19^b^	0.15–0.24
Social activity^c^ (Ref. = No)	0.57^b^	0.51–0.63	0.74^b^	0.66–0.84	0.25^b^	0.20–0.30	0.40^b^	0.32–0.49
Comorbidities (Ref. = No)								
Hypertension	1.09	0.99–1.20	1.00	0.89–1.11	1.24^b^	1.08–1.42	0.89	0.75–1.05
Diabetes mellitus	1.21^b^	1.07–1.36	1.18^b^	1.04–1.34	1.80^b^	1.55–2.10	1.82^b^	1.51–2.20
Stroke	2.54^b^	2.08–3.10	2.53^b^	2.04–3.14	6.86^b^	5.59–8.41	7.40^b^	5.71–9.59
Head injury	1.46^b^	1.14–1.86	1.60^b^	1.24–2.08	2.10^b^	1.57–2.80	2.24^b^	1.55–3.24
Cancer	0.86	0.67–0.10	0.95	0.73–1.23	1.38^b^	1.04–1.85	1.49^b^	1.04–2.12

CI, confidence interval; OR, odds ratio; Ref., reference.

^a^Estimated from multivariate logistic regression with levels of urbanization, sex, age, education year, lifestyle habits (smoking, drinking, regular exercise, social activity), and comorbidities (hypertension, diabetes mellitus, stroke, head injury, cancer) simultaneously included in the model.

^b^*P* < 0.05.

^c^To test the interaction terms for social activity with other lifestyle factors. For MCI, social activity has significant interaction with exercise (*P* = 0.008), but not with smoking (*P* = 0.75) and alcohol intake (*P* = 0.05). As for dementia, there was no significantly interactive effect of social activity with smoking (*P* = 0.12), alcohol intake (*P* = 0.65), and exercise (*P* = 0.24).

We further evaluated the ORs of these associated factors on MCI and dementia in urban, suburban, and rural areas, separately (Table 4). The results showed that women had a significantly higher prevalence of MCI (OR 1.40; 95% CI, 1.14–1.72) and dementia (OR 1.70; 95% CI, 1.24–2.33) than men in rural areas, but no gender difference was found in urban areas for the ORs of MCI and dementia. Regardless of any urbanization level, regular exercise has strong benefits for both MCI and dementia. The beneficial effects of exercise on dementia were much more evident in suburban (OR 0.15; 95% CI, 0.10–0.24) and rural areas (OR 0.13; 95% CI, 0.09–0.19). The benefits of social activity on dementia were similar between urban, suburban, and rural areas with ORs around 0.4. Regarding specific comorbidities, the impact of diabetes mellitus on dementia was significant in suburban (OR 1.96; 95% CI, 1.42–2.70) and rural area (OR 1.93; 95% CI, 1.45–2.57). Also, head-injury associated risk for MCI was significant in suburban and rural areas but not in urban areas, while the ORs of head injury on dementia were significant in suburban and urban areas but not in rural areas. For those who had a history of cancer, higher ORs of dementia were found only in rural areas (OR 1.99; 95% CI, 1.13–3.53).

**Table 4.** Adjusted odds ratios of the risk factors for mild cognitive impairment and dementia by levels of urbanization

	Mild Cognitive Impairment						Dementia					
	
	Urban		Suburban		Rural		Urban		Suburban		Rural	
					
	OR^a^	95% CI	OR^a^	95% CI	OR^a^	95% CI	OR^a^	95% CI	OR^a^	95% CI	OR^a^	95% CI
Sex												
Men	1.00		1.00		1.00		1.00		1.00		1.00	
Women	1.14	0.87–1.50	1.16	0.93–1.45	1.40^b^	1.14–1.72	0.92	0.60–1.41	1.62^b^	1.14–2.30	1.70^b^	1.24–2.33
Age, years												
65–69	1.00		1.00		1.00		1.00		1.00		1.00	
70–74	0.99	0.70–1.40	1.21	0.90–1.63	1.04	0.80–1.35	0.94	0.36–2.45	1.28	0.68–2.39	0.79	0.50–1.24
75–79	1.36	0.96–1.93	1.66^b^	1.23–2.24	1.59^b^	1.22–2.07	3.34^b^	1.45–7.69	3.15^b^	1.75–5.67	1.58^b^	1.02–2.44
80–84	1.62^b^	1.11–2.36	2.29^b^	1.67–3.15	2.17^b^	1.65–2.86	8.59^b^	3.87–19.07	5.73^b^	3.18–10.33	4.03^b^	2.26–6.17
≥85	2.21^b^	1.51–3.23	4.65^b^	3.28–6.60	3.62^b^	2.57–5.09	18.98^b^	8.69–41.43	27.94^b^	15.44–50.55	12.88^b^	8.09–20.51
Education, years												
0	2.61^b^	1.68–4.03	4.24^b^	2.64–6.81	3.46^b^	1.68–7.12	1.84	0.90–3.73	2.70^b^	1.25–5.84	3.38	0.96–11.97
≤6	1.33	0.89–1.98	2.28^b^	1.43–3.61	1.37	0.67–2.81	1.57	0.83–2.96	2.44^b^	1.14–5.22	1.76	0.50–6.21
7–12	0.89	0.58–1.37	1.54	0.94–2.54	0.79	0.35–1.76	1.36	0.69–2.67	1.09	0.47–2.50	1.52	0.40–5.80
>12	1.00		1.00		1.00		1.00		1.00		1.00	
Lifestyle habits												
Smoking (Ref. = No)	1.08	0.78–1.50	1.02	0.77–1.34	1.20	0.93–1.57	0.75	0.44–1.27	0.76	0.47–1.22	1.01	0.66–1.54
Drinking (Ref. = No)	1.03	0.71–1.50	1.17	0.87–1.58	0.82	0.60–1.11	1.32	0.73–2.39	0.83	0.48–1.43	0.51^b^	0.29–0.90
Regular exercise (Ref. = No)	0.67^b^	0.53–0.84	0.60^b^	0.50–0.73	0.66^b^	0.55–0.78	0.36^b^	0.24–0.54	0.15^b^	0.10–0.24	0.13^b^	0.09–0.19
Social activity (Ref. = No)	0.58^b^	0.46–0.74	0.86	0.70–1.05	0.77^b^	0.64–0.92	0.44^b^	0.29–0.66	0.43^b^	0.29–0.64	0.38^b^	0.27–0.53
Comorbidities (Ref. = No)												
Hypertension	0.92	0.73–1.15	1.07	0.89–1.29	0.99	0.84–1.17	0.86	0.59–1.25	0.98	0.73–1.31	0.82	0.63–1.06
Diabetes mellitus	1.18	0.91–1.54	1.42^b^	1.15–1.77	1.00	0.82–1.23	1.50	0.99–2.28	1.96^b^	1.42–2.70	1.93^b^	1.45–2.57
Stroke	2.46^b^	1.66–3.65	2.33^b^	1.58–3.42	2.76^b^	1.94–3.93	6.11^b^	3.64–10.25	8.71^b^	5.58–13.59	7.54^b^	4.95–11.49
Head injury	0.88	0.54–1.41	2.51^b^	1.63–3.89	1.98^b^	1.23–3.20	2.15^b^	1.19–3.89	2.68^b^	1.39–5.15	1.80	0.89–3.65
Cancer	0.92	0.56–1.52	0.91	0.59–1.41	1.04	0.66–1.61	0.94	0.47–1.87	1.36	0.73–2.54	1.99^b^	1.13–3.53

CI, confidence interval; OR, odds ratio; Ref, reference.

^a^Estimated from multivariate logistic regression with sex, age, education year, lifestyle habits (smoking, drinking, regular exercise, social activity), and comorbidities (hypertension, diabetes mellitus, stroke, head injury, cancer) simultaneously included in the model.

^b^*P* < 0.05.

To test the effects of aforementioned factors on prevalence of MCI or dementia vary across urbanization levels, we compared the odds ratios of each factor using a test for heterogeneity (eTable 2). For MCI, heterogeneity for age ≥85 (I^2^ = 75.46%, *P* = 0.017), social activity (I^2^ = 67.64%, *P* = 0.046), diabetes mellitus (I^2^ = 62.10%, *P* = 0.067), and head injury (I^2^ = 81.67%, *P* = 0.004) was high, reflecting the significant difference of the effects by these variables between urban, suburban, and rural areas. For dementia, we noted the effects of sex (I^2^ = 64.69%, *P* = 0.059), drinking (I^2^ = 61.25%, *P* = 0.076), and regular exercise (I^2^ = 85.53%, *P* = 0.001) were significantly different between levels of urbanization.

## DISCUSSION

This nationally representative survey is one of the few studies reporting the prevalence of MCI and dementia by urbanization levels in Asia to the best of our knowledge. It demonstrated the significant independent effect of urbanization on MCI and dementia prevalence in community-dwelling older people, with a more substantial magnitude for areas with lower urbanization levels. Besides, the impacts of various risk factors, such as sex, education year, and comorbidities, on the prevalence of MCI and dementia differed between levels of urbanization.

### MCI and dementia prevalence varied by urbanization levels

Our study findings were consistent with some previous results suggesting a higher prevalence of MCI^4^^–^^7^^,^^9^ and dementia^7^^–^^11^^,^^14^^–^^16^ in rural areas. The interpretation of this phenomenon could be multifaceted. First, rural living was associated with lower educational attainment^4^^,^^8^^,^^35^ and lower intellectually-demanding occupation,^4^ which may limit the development of cognitive reserve and lead to declining brain function.^36^ Second, rural residents tended to be delayed in screening and diagnosing chronic illness, possibly due to limited access to health-care services.^37^^,^^38^ Furthermore, poor management of chronic illness for rural residents may increase the likelihood of cognitive impairment.^39^ Third, certain lifestyle factors, such as physical activity^18^ and social participation,^17^ are usually more prevalent in urban regions, which contribute to the improvement of the cognitive reserve^36^ and mental health^40^^,^^41^ for urban residents.

Our study showed an independent effect of urbanization on MCI and dementia prevalence, even after adjustment for education, lifestyle, and health status. There may exist some other rural-urban related factors in association with the development of cognitive impairment. For example, pesticides are commonly used in rural areas.^24^ Animal studies have shown that animals exposed to pesticides may contribute to oxidative stress, α-synuclein fibrillization, mitochondrial dysfunction, and neuronal loss,^42^ ultimately leading to dementia in later life. Therefore, a higher prevalence of MCI and dementia in rural areas could be confounded by pesticide exposure.

### Rural-urban differences in the risk factors of MCI and dementia

Our findings revealed that the women had significant higher prevalence of dementia in suburban and rural areas instead of in urban region, which was compatible with a study in China.^8^ A possible explanation for this result is that rural women usually had lower education levels and lower mentally demanding jobs than men did in Taiwan. Substantial heterogeneity and uncertainty exist in the observed associations between alcohol consumption and dementia.^43^^,^^44^ In overall, drinking alcohol was not an independent factor for dementia or MCI in this study. The reason of the result about the association of alcohol drinking with reduced prevalence of dementia only in rural areas is not clear. Since alcohol-related deaths are higher in rural area than in urban ones in Taiwan,^45^ we suspected our results may be much explained by survival bias in rural areas.^46^

Although the heterogeneity estimates showed no different among the urbanization statuses regarding the relation of diabetes mellitus with dementia, this vascular risk factor was significantly associated with higher prevalence of dementia than participants without diabetes in suburban and rural areas. A study with three representative cohorts in Taiwan revealed that rural diabetic patients were less likely to receive guideline-recommended tests; thus, increasing the likelihood of avoidable hospitalizations than their urban counterparts.^47^ Poor adherence to recommended tests leads to acute or chronic hyperglycemia and the risk of events of severe hypoglycemia. Inadequate sugar control, either with chronic high blood sugar or severe hypoglycemia episodes, significantly increased the risk of dementia.^48^^,^^49^

An epidemiological analysis in Taiwan showed that hospitalization rate and severity of head injury were higher in rural areas than in urban residency.^50^ The likelihood of developing post-trauma cognitive impairment is related to the severity of brain injury, which may explain that the head injury survivors have a higher risk of MCI in suburban and rural areas in this study. Besides, the in-hospital mortality of moderate-to-severe head injury was significantly higher in rural residents than in their urban counterparts.^50^ Therefore, our results of the higher proportion of head-injury survivors with dementia in urban and suburban areas than in rural areas may probably be due to the survival bias that distorts the epidemiological relationships between urbanization and the risk of dementia.

The association between cancer and the risk of dementia is inconclusive. Some studies reported cancer as a risk factor for cognitive impairment and dementia, while some studies suggested particular cancer reduced the risk of dementia, even with consideration of survival bias.^51^^–^^53^ In the present study, we found that cancer increased dementia in rural populations but not in urban ones. These findings might be attributed to the rural-urban disparity in cancer type, which needs to be further confirmed.

### Methodological concerns and conclusion

This study has several strengths. First, this research is a nationwide representative community survey to explore the roles of urbanization in the risk of MCI and dementia in older people. Second, this study also compared urban, suburban, and rural areas regarding MCI and dementia’s contributed factors, which has been rarely conducted before.^8^ Third, since previous study showed that less accessibility of medical services in rural areas may lead to the MCI and dementia underestimated,^13^ our study used a door-to-door community-based case-finding strategy, making it possible to find cases that would have never been presented to a doctor, particularly the MCI cases in rural areas.

This study also has several limitations as followings. The first is the low response rate (36.5%) in this national survey.^25^ Institutionalization due to dementia was one of the causes for non-responding and the proportions of institutionalization were probably different between urbanization levels. Nevertheless, the institutionalized non-respondents (*n* = 238) accounted for only 0.8% of sampled population, which may not significantly affect the overall results. In addition, there was no significant difference regarding the distribution of gender and age between non-respondents and respondents in two selected counties, in which one is highly urbanized while another one is comprised of more suburbs and rural areas.^25^ Despite that, we cannot assume that the distribution of dementia and MCI between urbanization levels in non-respondents was similar to that in participants, and there still remained residual selection bias. Second, this cross-sectional study demonstrated that the association of urbanization with MCI and dementia in the elderly is influenced by sociodemographic, lifestyle habits, and health factors. However, the causal effects of these factors and the role of cumulative residence could not be evaluated without longitudinal data. Third, owing to the lack of information on dementia types, we could not distinguish the subtypes of dementia, limiting further interpretation of study results. Fourth, some potential environmental factors, such as pollutants and pesticide use, may play some roles in the association between urbanization and the development of MCI and dementia. These potential confounders were not available and could not be adjusted in this study.

In conclusions, this door-to-door national survey revealed that older people from rural areas had a higher prevalence of MCI and dementia than those in urban areas. Sex, education year, and specific health factors have different impacts between rural and urban residents on MCI and dementia prevalence. Specific interventions for dementia prevention for rural residents should be considered in future public health policy to reduce the rural-urban inequality.

## SUPPLEMENTARY MATERIAL

eTable 1. Sex- and age-specific prevalence of mild cognitive impairment and dementia by levels of urbanization, Taiwan, 2011–2013

eTable 2. Heterogeneity estimates of each risk or protective factor for mild cognitive impairment and dementia among the urbanization levels, Taiwan, 2011–2013
