# Supplementary material for: Rural-urban Disparities in the Prevalence of Mild Cognitive Impairment and Dementia in Taiwan: A Door-to-door Nationwide Study
Source: J Epidemiol. 2022 Nov 5;32(11):502–9. doi: 10.2188/jea.JE20200602 (PMC9551297; doi:10.2188/jea.JE20200602)
Supplement: Supplementary file 1 [file je-32-502-s001.pdf]

eTable 1. Sex- and age-specific prevalence of mild cognitive impairment and dementia by levels of urbanization, Taiwan, 2011–2013

Cognitive status<br>Sex<br>Age, years	Sex- and Age-specific Rates, % (95% CI)[a]			Rural / Urban Ratio
	Urban	Suburban	Rural	
Mild cognitive impairment				
Men				
65–69	10.60 (10.32–10.88)	9.93 (9.73–10.13)	11.25 (11.06–11.44)	1.06 (0.63–1.79)
70–74	10.57 (10.39–10.76)	13.08 (12.95–13.21)	15.13 (15.00–15.27)	1.43 (0.96–2.13)
75–79	14.98 (14.70–15.26)	16.79 (16.61–16.98)	19.09 (18.95–19.24)	1.27 (0.87–1.86)
80–84	17.41 (17.08–17.74)	22.40 (22.14–22.66)	21.27 (21.06–21.47)	1.22 (0.84–1.79)
≥85	21.33 (20.98–21.69)	25.60 (25.26–25.94)	23.92 (23.52–24.32)	1.12 (0.75–1.67)
Women				
65–69	14.98 (14.74–15.23)	14.01 (13.82–14.20)	19.74 (19.53–19.94)	1.32 (0.91–1.92)
70–74	15.49 (15.30–15.67)	17.83 (17.70–17.95)	22.76 (22.68–22.89)	1.47 (1.08–1.99)
75–79	21.97 (21.72–22.23)	24.57 (24.39–24.75)	29.75 (29.58–29.92)	1.35 (1.02–1.80)
80–84	20.10 (19.71–20.48)	22.73 (22.46–22.99)	32.42 (32.19–32.65)	1.61 (1.14–2.29)
≥85	19.59 (19.19–19.99)	25.91 (25.52–26.30)	34.11 (33.68–34.55)	1.74 (1.18–2.58)
Dementia				
Men				
65–69	2.30 (2.17–2.44)	2.74 (2.63–2.85)	5.63 (5.48–5.77)	2.44 (0.91–6.58)
70–74	1.51 (1.44–1.58)	2.62 (2.55–2.68)	2.68 (2.62–2.74)	1.78 (0.64–4.93)
75–79	4.45 (4.29–4.62)	6.02 (5.90–6.13)	5.29 (5.21–5.38)	1.19 (0.59–2.39)
80–84	10.71 (10.44–10.98)	7.57 (7.41–7.73)	12.41 (12.24–12.57)	1.16 (0.71–1.89)
≥85	17.78 (17.44–18.11)	18.80 (18.49–19.11)	19.14 (18.77–19.51)	1.08 (0.69–1.67)
Women				
65–69	1.05 (0.98–1.11)	2.52 (2.43–2.61)	5.26 (5.15–5.38)	5.04 (1.50–16.94)
70–74	1.31 (1.25–1.37)	3.74 (3.67–3.80)	6.89 (6.81–6.97)	6.59 (2.61–16.64)
75–79	4.46 (4.33–4.59)	9.70 (9.57–9.82)	10.75 (10.63–10.87)	2.41 (1.34–4.33)
80–84	11.76 (11.46–12.07)	16.23 (16.00–16.47)	17.46 (17.27–17.64)	1.48 (0.93–2.36)
≥85	21.13 (20.72–21.55)	37.73 (37.30–38.16)	43.93 (43.47–44.38)	2.08 (1.44–3.00)

CI, confidence interval.

[a] Prevalence rates were calculated per 100 persons.

eTable 2. Heterogeneity estimates of each risk or protective factor for mild cognitive impairment and dementia among the urbanization levels, Taiwan, 2011–2013

Variables	Mild Cognitive Impairment			Dementia		
	Cochran Q	*P* value	I^2	Cochran Q	*P* value	I^2
Sex (Ref.=Men)	1.93	0.38	0.00	5.66	0.06	64.69[a]
Age, years (Ref.= 65–69)						
70–74	0.95	0.62	0.00	1.50	0.47	0.00
75–79	0.78	0.68	0.00	4.61	0.10	56.61
80–84	2.15	0.34	6.92	2.95	0.23	32.19
≥85	8.15	0.02	75.46[a]	4.10	0.13	51.20
Education Years (Ref.= >12)						
0	2.21	0.33	9.49	0.91	0.63	0.00
≤6	3.24	0.20	38.22	0.78	0.68	0.00
7–12	3.33	0.19	39.93	0.24	0.89	0.00
Lifestyle habits (Ref.=No)						
Smoking	0.77	0.68	0.00	1.04	0.60	0.00
Drinking	2.70	0.26	25.96	5.16	0.08	61.25[a]
Regular exercise	0.63	0.73	0.00	13.8	0.00	85.53[a]
Social activity	6.18	0.05	67.64[a]	0.34	0.84	0.00
Comorbidities (Ref.=No)						
Hypertension	1.05	0.59	0.00	0.79	0.67	0.00
Diabetes mellitus	5.41	0.07	62.10[a]	1.18	0.56	0.00
Stroke	0.43	0.81	0.00	1.04	0.60	0.00
Head injury	10.91	0.00	81.67[a]	0.66	0.72	0.00
Cancer	0.19	0.91	0.00	2.75	0.25	27.38

Ref, reference.

[a] $P < 0.1$
